# Recurrent generalized seizures as the prominent manifestation in a patient with CADASIL: a case report and literature review

**DOI:** 10.1186/s12883-022-02889-7

**Published:** 2022-09-30

**Authors:** Liuhua Pan, Yan Chen, Shanshan Zhao

**Affiliations:** Department of Neurology, the First Affiliated Hospital of China Medical University, Number 155, Nanjing Street, Heping District, Shenyang City, 110001 Liaoning Province China

**Keywords:** CADASIL, Generalized seizures, NOTCH3 gene, Review, Case report

## Abstract

**Background:**

Cerebral autosomal dominant arteriopathy with subcortical infarcts and leukoencephalopathy (CADASIL) is an inherited arteriopathy typically caused by mutations in the NOTCH-3 gene. Few detailed descriptions of recurrent generalized seizures in CADASIL has been reported.

**Case presentation:**

This article details a case of recurrent generalized seizures, which eventually be diagnosed as CADASIL with a heterozygous variant, c.1630 C > T (p. Arg544Cys), in exon 11 of the Notch 3 gene. Here, we discussed the possible pathogenesis underlying the epilepsy associated with CADASIL through the brain magnetic resonance imaging changes and the captured epileptiform waves in the electroencephalography during the patient’s follow-up period. Related literatures were also reviewed to discuss the etiology of the epilepsy.

**Conclusions:**

Recurrent generalized seizures may be a presenting neurological manifestation of CADASIL in the absence of other discernible causes. Clinicians should comprehensively seek the possible etiology of patients with recurrent generalized seizures, considering the possible diagnosis of CADASIL.

## Background

Cerebral autosomal dominant arteriopathy with subcortical infarcts and leukoencephalopathy (CADASIL) is a non-amyloid and non-arteriosclerotic type of monogenic hereditary cerebral small vessel disease. CADASIL usually occurs in middle age, with some characteristic clinical features, such as migraine with aura, recurrent stroke, transient ischemic attack, mood disturbances, and progressive cognitive impairment [1]. Approximately 10% of CADASIL patients develop epileptic seizures that related with ischemic stroke [2, 3]. Seizure is rarely the first onset symptom and primary manifestation of CADASIL [3], and the exact cause of seizures is not clear yet. Here we describe a Chinese woman with CADASIL carrying the Arg544Cys mutation manifesting with recurrent generalized seizures as the prominent clinical phenotype. We reviewed the clinical and neuroimaging characteristics of this patient along with relevant literatures and discussion.

## Case presentation

A 54-year-old right-handed woman was admitted in our hospital for new-onset epilepsy in May 2018. One month before admission, the patient felt dizzy while watching TV, followed by her left limb twitching, without unconsciousness. Then she experienced a 3-min generalized tonic–clonic seizure (GTCS) with unconsciousness. There were totally three episodes of generalized seizures running for several minutes each that day. Seizures relieved and consciousness gradually recovered after intravenous administration of diazepam. However, the numbness and weakness in her left limbs were heavier than usual. Initially, her symptoms were considered due to "acute cerebral infarction with epileptic seizures" by the local hospital. So she received aspirin, edaravone and other routine care of stroke. On the meanwhile, sodium valproate was commenced 500 mg/day orally to control seizure. Then the patient’s left limbs numbness and weakness were alleviated, and the seizure did not recur again. But the patient's cognitive function became worse and began to influence her daily life from then on, so they came to our hospital for further evaluation and treatment.

The patient denied any previous history of seizures, head trauma, and central nervous system infection. Although without any traditional vascular risk factors, she had an ischemic stroke 4 years ago, leaving numbness and slight weakness both in her left upper and lower limb. Her recent memory started to decline one year ago. Her father and mother had died with no cerebrovascular disease in the age about 70 to 80 years old. But her younger brother had a cerebral infarction around the age of 30.

**Fig. 1:**
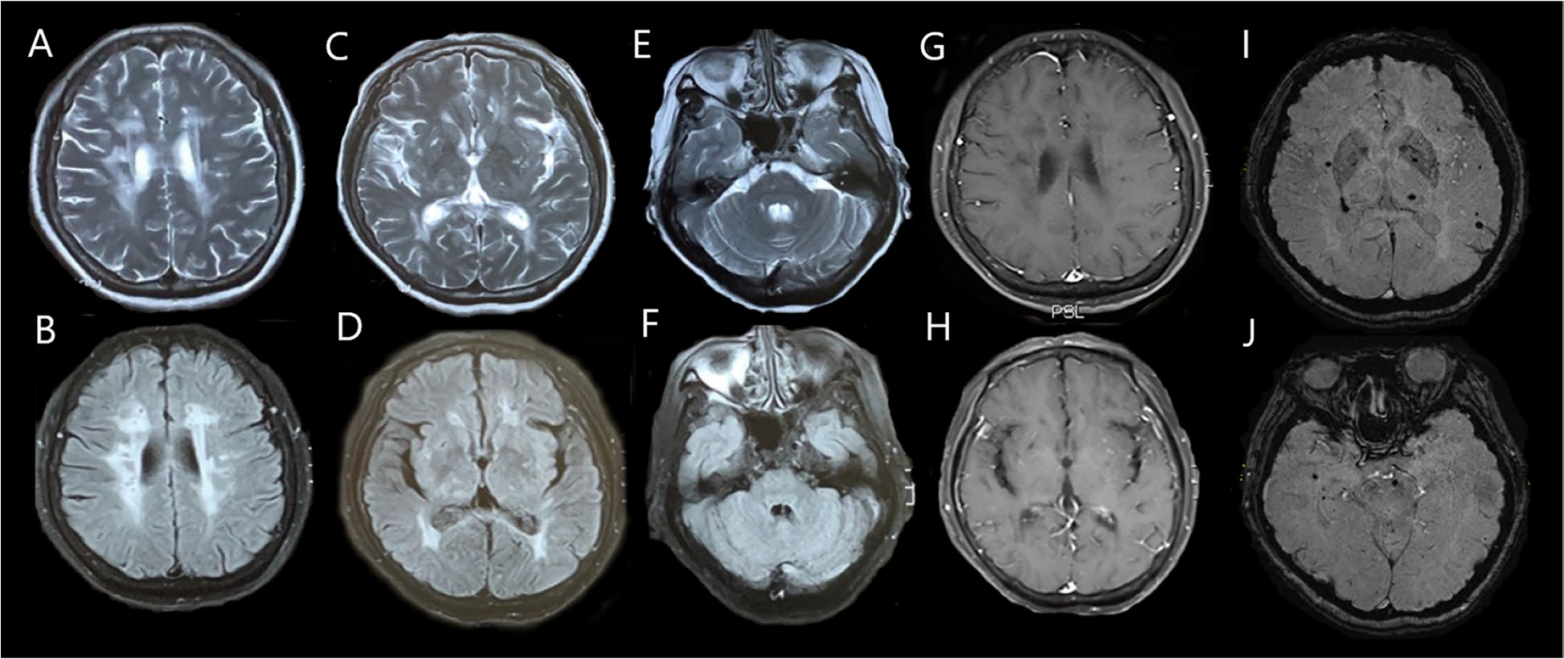
Brain MRI of the patient in May 2018T2-weighted (**A**, **C**, **E**) and FLAIR images (**B**, **D**, **F**) showed multiple lacunar infarcts throughout the bilateral basal ganglia, thalamus, periventricular white matter and subcortical white matter. Enhanced scan showed no obvious enhancement (**G**, **H**). Susceptibility-weighted imaging showed the multiple cerebral microbleeds in the basal ganglia, brain stem and subcortical areas (**I**, **J**)

**Fig. 2:**
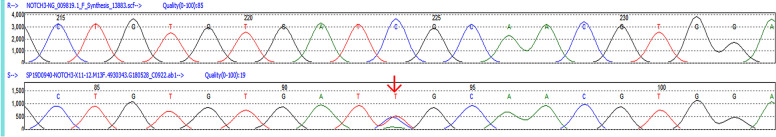
The mutation analysis of the NOTCH3 gene CADASIL pathogenic gene NOTCH3 mutation hot zone (3-6/11-14/18-19) sequencing confirmed a heterozygous mutation, exon 11 c.1630C > T p (Arg544Cys). (Top: Reference sequence; bottom: the patient’s genetic analysis)

In the physical examination, the patient was normal in the mental status but responded slowly to the doctor’s commands. She had a weakness (muscle strength IV grade) and decreased sensation to light touch and pain in her left limbs. She also showed tendon hyperreflexia and positive Babinski sign in her left lower limb. After admission, her video-electroencephalogram showed slow waves with middle-amplitude from right middle anterior temporal lead, and suspicious sharp-slow waves which are greater on the right anterior frontal lead. Brain magnetic resonance imaging (MRI) demonstrated extensive high T2 and fluid attenuated inversion recovery (FLAIR) signals in bilateral basal ganglia, periventricular white matter and subcortical white matter (Fig. 1 a-f), but no obvious enhancement was showed (Fig. 1 g, h). Multiple microbleeds were detected with susceptibility-weighted imaging (Fig. 1 i, j). Transcranial doppler, carotid ultrasound, echocardiograms, 24-h dynamic electrocardiogram and cerebrospinal fluid assessment were all normal. The autoimmune antibodies associated with paraneoplastic and autoimmune encephalitis, and the oligoclonal bands in serum and cerebrospinal fluid were both negative. The results of routine laboratory tests were unremarkable. Finally, she was suspected of the diagnosis of CADASIL based on the neuroimaging and the personal and family histories. This suspicion was confirmed by genetic analysis, showing a heterozygous missense mutation c.1630 C > T, which resulting in p. Arg544Cys, in exon 11 of Notch 3 gene (Fig. 2).

**Fig. 3:**
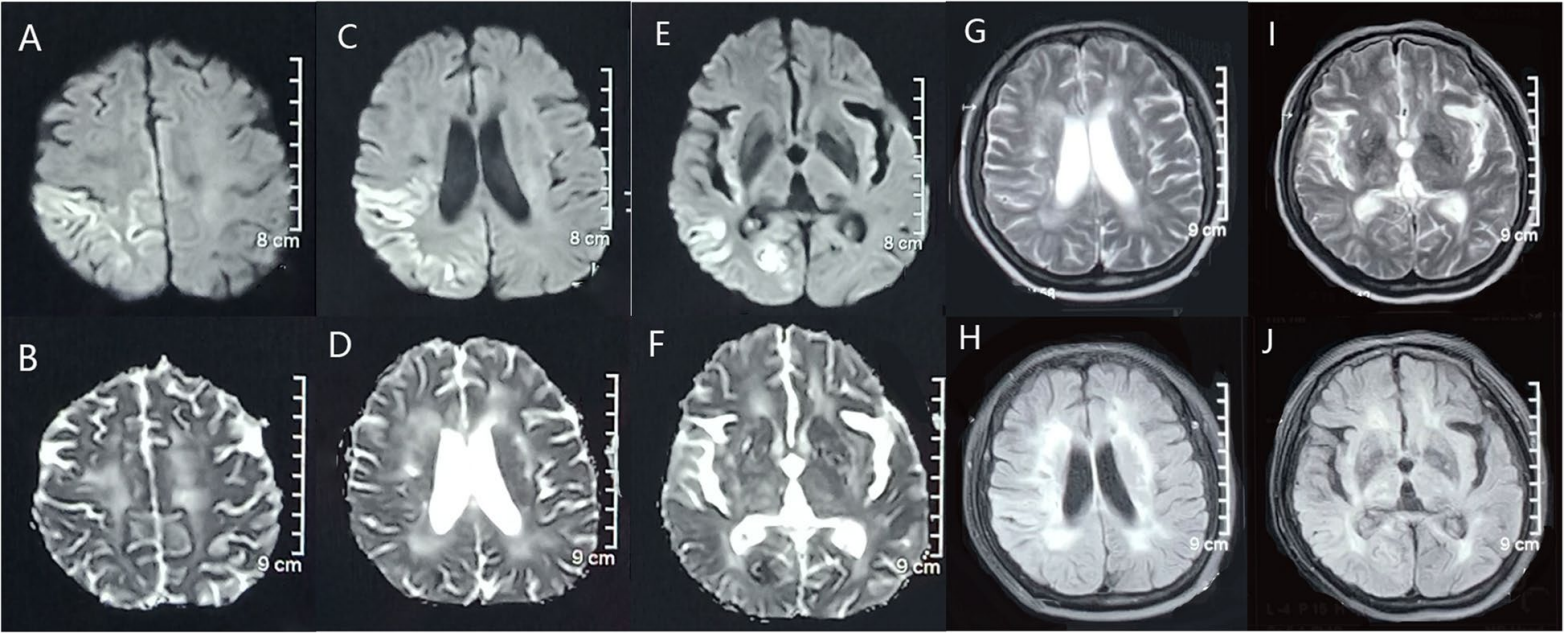
Brain MRI of the patient in August 2019 DWI showed the high signal in the cortices of right parietal lobe, right temporal lobe and right occipital lobe (**A**, **C**, **E**) with corresponding decreased ADC values (**B**, **D**, **F**). Brain MRI showed multiple ischemic foci and softened foci on T2 image (**G**, **I**), and extensive white matter lesions on FLAIR image (**H**, **J**)

In August 2019, sudden generalized seizures happened to her again. Brain MRI still showed multiple cortical and subcortical ischemic infarcts, softening foci, and leukoaraiosis on T2 and FLAIR images (Fig. 3 G-J), which were more serious than the findings in 2018. Particularly, diffusion-weighted imaging(DWI)showed high signal in right parietal, temporal and occipital lobe (Fig. 3A,C and E) with corresponding areas of decreased signal on apparent diffusion coefficient (ADC) map (Fig. 3B, D and F).

**Fig. 4:**
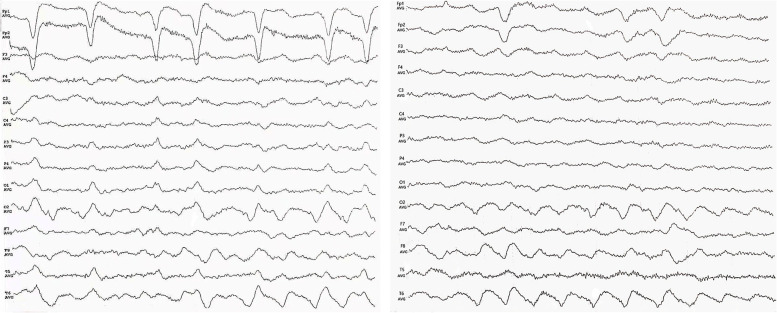
The electroencephalogram of the patient in November 2019 Limited 1-3c/s slow waves over the right temporo-occipital regions and sharp-slow waves over the occipital region during awake (left); limited 1.5-6c/s slow waves over the right temporo-occipital region during sleep (right)

**Fig. 5:**
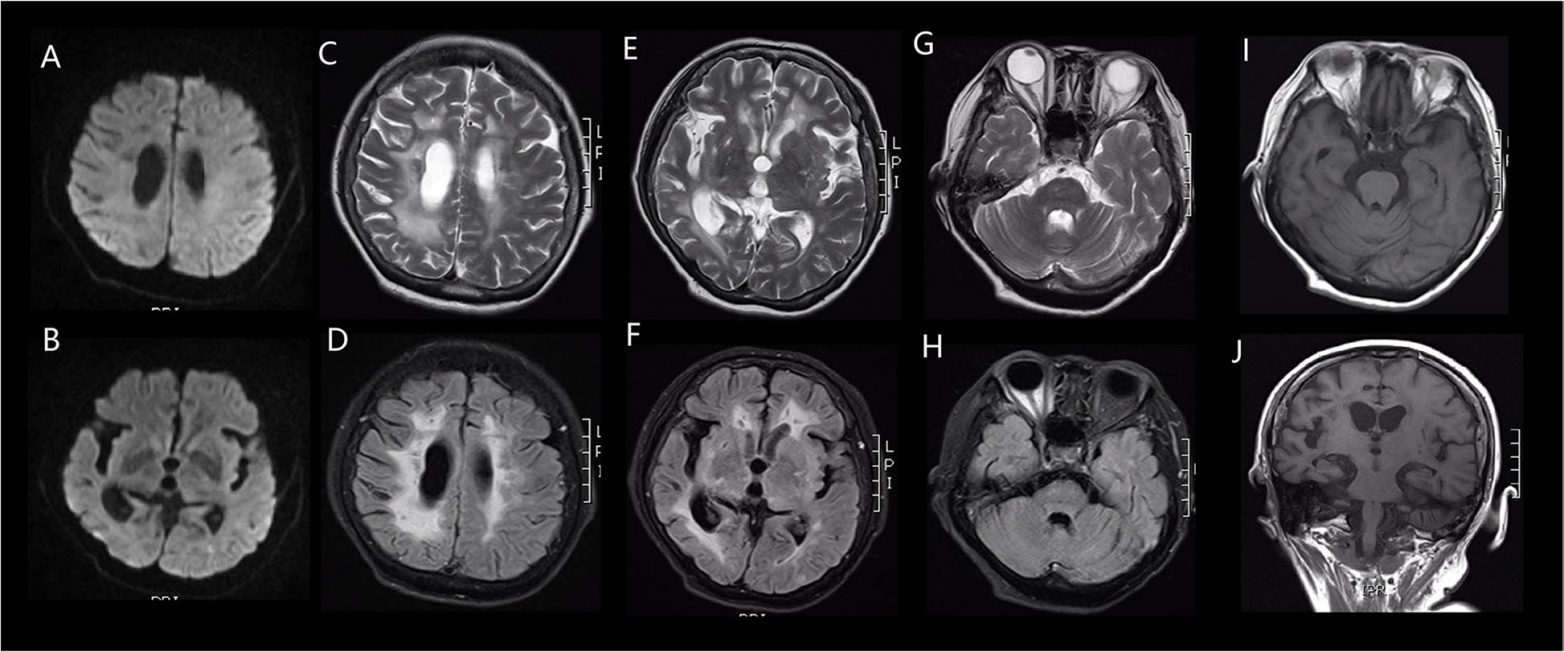
Brain MRI of the patient in November 2019 DWI showed no recent infarctions (**A**, **B**). MRI showed high T2 (**C**,**E**,**G**) and FLAIR (**D**, **F**, **H**) signals of multiple cortical and subcortical ischemic infarcts, softening foci, and extensive confluent white matter lesions. There is also global atrophy, particularly in the bilateral hippocampus (**I**, **J**) and the right temporo-apical lobe (**C**-**F**)

In November 2019, she was transferred to our hospital again with the diagnosis of “convulsive status epilepticus”. Interictal EEG still recorded sharp and slow waves in right temporal-occipital leads (Fig. 4). MRI also showed brain atrophy including bilateral hippocampal atrophy, but without any acute ischemic lesions (Fig. 5). Because frequent seizures continued to happen, oxcarbazepine was added. Several days later, the epilepsy was relieved, but the patient started sustained nonsense, inability to answer questions correctly, and having no sleep at night. Therefore, phenobarbital and clonazepam were added, and the levetiracetam was stopped. Then the patient alleviated gradually and was discharged. After that she continued to take oxcarbazepine and phenobarbital, as well as antithrombotic therapy such as aspirin, rosuvastatin and ezetimibe. However, generalized tonic–clonic seizures still happened intermittently.

## Discussion and conclusions

CADASIL is an adult-onset, autosomal dominant inherited cerebral small vessel disease and the most common etiological diagnosis of hereditary cerebrovascular disease [4]. In 1996, Joutel A et al. confirmed that the disease was caused by mutations in the NOTCH3 gene located on the short arm of chromosome 19 [5] and genetic testing is currently considered to be the gold standard for the diagnosis of CADASIL [4]. CADASIL is associated with a variety of symptoms, among which epilepsy is a rare condition. In an early report on the phenotypic spectrum and natural history of the disease in 102 patients with CADASIL, only 10 patients had seizures, most of which were generalized tonic–clonic seizures and occurred after the onset of stroke. Considering that most of these patients with epileptic seizures had a history of stroke, and none of them had epilepsy as the prominent phenotype, the author tended to support that seizures were secondary to ischemic lesions in CADASIL patients [3]. In addition, ischemic lesions have been considered to be an important risk factor for late-onset epilepsy in patients with sporadic stroke [3]. There are few reports describing recurrent generalized seizures as the prominent clinical manifestations of CADASIL.

Here we report a case of a middle-aged female patient with CADASIL who manifested recurrent generalized seizures, together with symptoms of mild focal neurological deficit, and these symptoms improved after receiving stroke treatment. Hence, firstly the possible origin of this episode was speculated to be correlated with an ischemic stroke by local hospital. Fortunately, we suspected of the diagnosis of CADASIL based on her characteristic neuroimaging, the cognitive function impairment and family histories. Finally, this suspicion was confirmed by genetic analysis.

In this case, the patient demonstrated focal to bilateral tonic–clonic seizure firstly, then recurrent generalized tonic–clonic seizures, and finally the convulsive status epilepticus. Her interictal EEGs showed the epileptic activities was either originated from right frontal–temporal leads firstly, or from temporal-occipital leads. We considered that the abnormal discharges might origin from the right temporal and occipital lobes, then spread to the ipsilateral frontal lobe and the contralateral cerebral hemisphere through electric field effects and transmission pathways, thus led to recurrent generalized tonic–clonic seizures. So the patient was diagnosed with focal epilepsy, whereas she had recurrent generalized seizures as prominent manifestation. This type of seizures can’t be controlled easily by the common antiepileptic drugs, which might due to the inconsistency between the type of seizure and the type of epilepsy, and need to be further investigated in more such patients.

Whether seizures are one of the manifestations of CADASIL or secondary to ischemic stroke events is still controversial. In brief, CADASIL has a pathological basis for seizures; epileptogenic cortical lesions can cause seizures in CADASIL patients, and the prevalence of epilepsy in CADASIL patients may also be underestimated. In a study on a CADASIL using high-resolution 7-T postmortem MRI combined with neuropathological examination of the brain, CADASIL patients showed a large number of tiny intracortical and subcortical infarcts [6], which are postulated as structural aetiology of epilepsy in CADASIL [7]. On the other hand, it has been previously reported that leukoaraiosis is often observed in adult patients with otherwise unexplained new-onset epilepsy (epilepsy associated with leukoaraiosis, EAL) [8, 9]. Gasparini et al. further found that temporal lobe epilepsy predominates in patients with EAL [9]. Ferlazzo et al. proposed that systemic hypertension and leukoaraiosis may regulate the susceptibility to epilepsy, and the coexistence of occult cortical microinfarction or other auxiliary factors may also lead to the tendency of seizures [8]. In this case, repeated MRIs all showed extensive leukoaraiosis in bilateral basal ganglia, periventricular white matter, which might be epileptogenic. There was only one cortical lesion on the MRI when the patient was admitted to our hospital with convulsive status epilepticus, which showed high DWI and low ADC signals in the right parietal, temporal and occipital lobe, but disappeared three months later, and there was no corresponding signal change on T2WI (Fig. 3). Given the lack of typical clinical manifestations of acute stroke, such as dysfunction of language, hearing, vision, etc., the cortical MRI changes here were probably due to transient changes caused by epilepticus seizures rather than stroke. Furthermore, Abraira's study reported that the hippocampal atrophy in patients with late-onset epilepsy was heavier than other populations, suggested that the neurodegenerative changes in the medial temporal lobe structure could be one of the predisposing factors for seizures [10]. Our patient’s repeated brain MRI showed aggravated brain atrophy during the disease course, that is consistent with this opinion.

The main imaging manifestations of CADASIL on brain MRI include white matter hyperintensities, lacunar infarctions, cerebral microbleeds, enlarged perivascular spaces, and brain atrophy [11]. As far as we know, white matter hyperintensities (WMH) of CADASIL initially affects the semi-oval center and then gradually extends to the bilateral temporal pole, external capsule, and callosum as the course progresses. It is worth noting that the presence of periventricular white matter hyperintensities has great significance; without it, the diagnosis of CADASIL is questionable. Our patient's MRIs showed clear extensive confluent white matter lesions in the periventricular area and the centrum semiovale on T2/ FLAIR image, which supports the diagnosis of CADASIL. On the other hand, characteristic anterior temporal white matter hyperintensity is also a typical imaging marker for the diagnosis of CADSIL, but the incidence of which in Asian patients is relatively low [12, 13]. The Asian female patient reported in our case did not demonstrate a characteristic anterior temporal lesion, which may be due to her specific geography or genotype.

Some scholars believe that specific NOTCH3 gene mutation sites are associated with clinical phenotypes [11]. Previous studies have found that the clinical phenotypes of patients with c.1630C > T (p. Arg544cys) variants include headache, stroke, mood disorders, cognitive decline, etc. [14–16], and there are few detailed reports of clinical phenotypes as recurrent generalized seizures. Oh JH reported a patient with p.R544C mutation and initially showed an episode of generalized tonic–clonic seizures, but this patient remained seizure-free after antiepileptic treatment [17]. This is different from our case with spontaneous recurrent generalized seizures as the prominent manifestation. In this case, the patient’s brother had a stroke in a very young age, indicating that he was possibly also affected by CADASIL. So the pathogenic mutation of this patient might not be a de novo mutation. Unfortunately, the other family members of the patient refused the further verification test. The genotypes reported in the rest literature of CADASIL presenting with epileptic seizures are scattered at other mutation sites in a seemingly random manner [18–20]. Moreover, it is showed that CADASIL gene mutation loci are associated with different clinical phenotypes [21], and the clinical phenotype and severity of CADASIL may be different even in the same family [22]. In future, the genotype–phenotype correlation analyzes of each mutation should be carried out with care due to the wide distribution of phenotypes, even in the same family that harbors the same mutation. Due to the small number of cases of CADASIL combined with recurrent generalized seizures, the correlation between NOTCH3 genotype and this clinical phenotype should be further confirmed. Besides, other genetic modifiers and factors other than the heredity, such as traditional vascular risk factors, environmental differences, treatment compliance, also play an important role in causing phenotypic variation in the natural course of the disease [23].

In summary, seizures are uncommon in CADASIL patients and can happen simultaneously with stroke, precede or after an attack of stroke. Our case indicated that recurrent generalized seizures may be a presenting neurological manifestation of CADASIL in the absence of other discernible causes. Therefore, extensive consideration about whether the disease is accompanied by some core clinical manifestations, such as headache, stroke, and cognitive impairment, as well as a genetic history should be given for epilepsy of unknown etiology. Once the brain MRI shows white matter hyperintensities, lacunar infarctions, cerebral microbleeds, and brain atrophy changes, the possible diagnosis of CADASIL should be taken into account, and the genetic test should be carried out if the patient has family history.

## Data Availability

We are unable to provide any additional clinical data in the interest of protecting the patient’s privacy.

## Abbreviations

CADASIL: Cerebral autosomal dominant arteriopathy with subcortical infarcts and leukoencephalopathy
GTCS: Generalized tonic–clonic seizure
MRI: Magnetic resonance imaging
FLAIR: Fluid attenuated inversion recovery
DWI: Diffusion weighted imaging
ADC: Apparent diffusion coefficient
EAL: Epilepsy associated with leukoaraiosis
